# Supplementary material for: Atomic model of vesicular stomatitis virus and mechanism of assembly
Source: Nat Commun. 2022 Oct 10;13:5980. doi: 10.1038/s41467-022-33664-4 (PMC9549855; doi:10.1038/s41467-022-33664-4)
Supplement: Supplementary file 1 — Supplementary information [file 41467_2022_33664_MOESM1_ESM.pdf]

SUPPLEMENTARY INFORMATION

Atomic model of Vesicular Stomatitis Virus and Mechanism of Assembly

Kang Zhou[1,2,3,#], Zhu Si[1,2,#], Peng Ge[2,&], Jun Tsao[4], Ming Luo[5], Z. Hong Zhou[1,2,*]

[1]Department of Microbiology, Immunology & Molecular Genetics, University of California, Los Angeles (UCLA), Los Angeles, CA 90095, USA

[2]California NanoSystems Institute, UCLA, Los Angeles, CA 90095, USA

[3]School of Life Science, University of Science and Technology of China, Hefei, Anhui 230026, P.R. China

[4]Department of Microbiology, University of Alabama at Birmingham, Birmingham, Al 35294, USA

[5]The Department of Chemistry, Georgia State University, Atlanta, GA 30303, USA

[#]These authors contributed equally.

Corresponding author email: Hong.Zhou@UCLA.edu; Phone: 310-694-7527

[&]**Current address:** Departments of Chemistry and Biochemistry and Biological Chemistry, and Howard Hughes Medical Institute, UCLA, Los Angeles, CA 90095, USA

Supplementary Figures

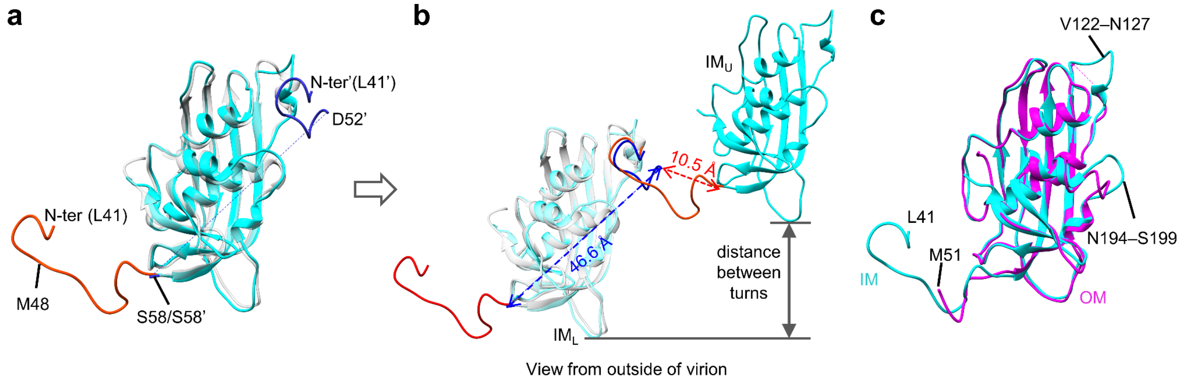

Supplementary Fig. 1 | Structure comparison of M. a: Superposition of the *in situ* structure of M (IM) and crystal structure of M. The *in situ* IM is colored in cyan except for the N-termini, in red, while the M in crystal structure is colored in transparent gray except for the N-termini, in blue. **b**: Distance between terminal residues of IM in cryo EM structure and the determination of N-termini affiliation. 7 residues (D52-S58) were missing in the structure. Between the blue N-terminal segment and the IM_U is a distance of 10.5 Å, which can be bridged by those 7 residues; between the same segment and the IM_L is a distance of 46.6 Å, which cannot. Therefore, the blue N-terminal segment must belong to IM_U. **c:** Superposition of the inner M (IM) and outer M (OM) subunits. IM is colored in cyan and OM in magenta.

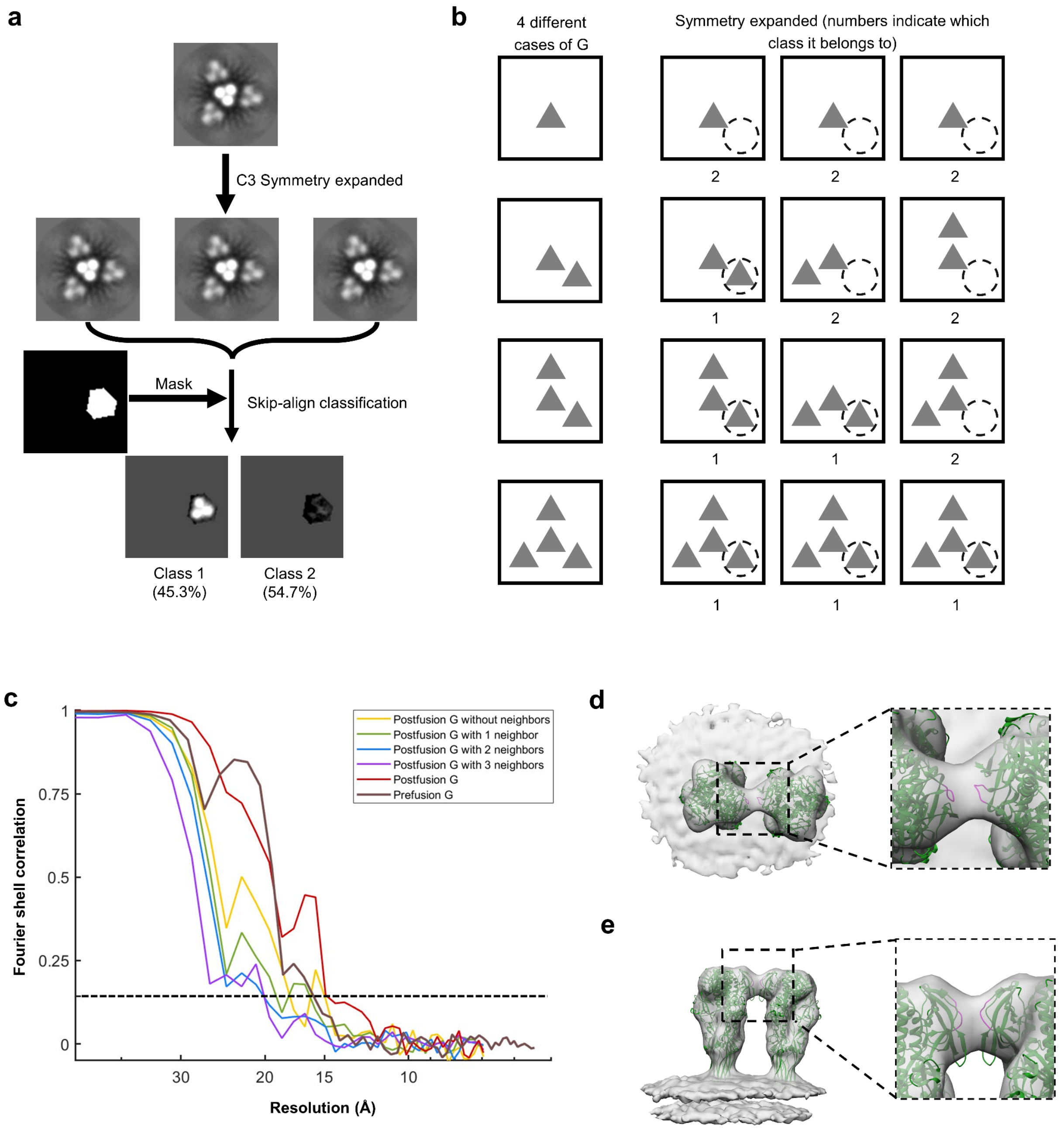

Supplementary Fig. 2 | CryoET analysis for VSV G. a, b: Workflow of 3D classification of G trimers with different numbers of neighboring G trimers. **c:** Fourier shell correlation coefficient as a function of spatial frequency of subtomogram averages of G with different numbers of neighboring G trimers. The dashed line depicts the 0.143 resolution cut-off. Red: average of postfusion G regardless of number of neighboring G trimers, 14.9 Å; yellow: average of postfusion G without neighboring G trimers, 18.4 Å; green: average of G with one neighboring G

trimer, 19.6 Å; blue: average of G with two neighboring G trimers, 20.9 Å; purple: average of G with all three neighboring G trimers, 20.9 Å; brown: average of prefusion G, 15.8 Å. **d, e:** Subtomogram averaged structure of G with one neighboring G trimer fitted with atomic models of the crystal structure of G in postfusion conformation, showing the interactions between two G trimers in top (**d**) and side views (**e**). N-termini of G (residues 8-14), colored magenta, are potentially involved in the interaction.

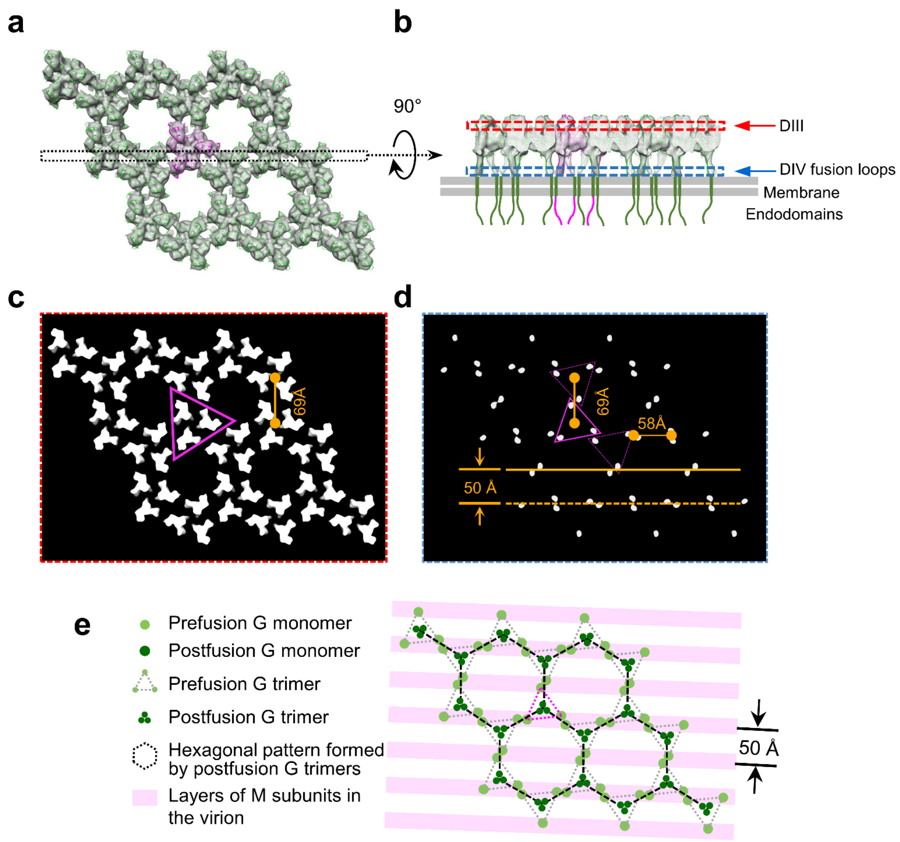

Supplementary Fig. 3 | 2D hexagonal lattice in crystals of prefusion G. a, b: Density maps derived from atomic models of prefusion G trimers showing the 2D hexagonal lattice in crystals. A density map is filtered to 15 Å. Models colored as magenta are one of the prefusion trimers. (**a**) is the top view; (**b**) is the side view. **c, d:** top views of the density map from different sections, indicated as arrows in (**b**) (red arrow for **c** viewing at DIII, blue arrow for **d** viewing at DIV fusion loops). The magenta triangle indicates the same prefusion trimer in (**a**) and (**b**). Distances between the DIV pairs, between the centers of two G trimers and the equilateral triangle height of the G trimer are measured as 58 Å, 69 Å and 50 Å, respectively. **e:** Illustration of the 2D lattice formed by G trimers on the viral membrane. The organization of G matches with that of OM inside the membrane.

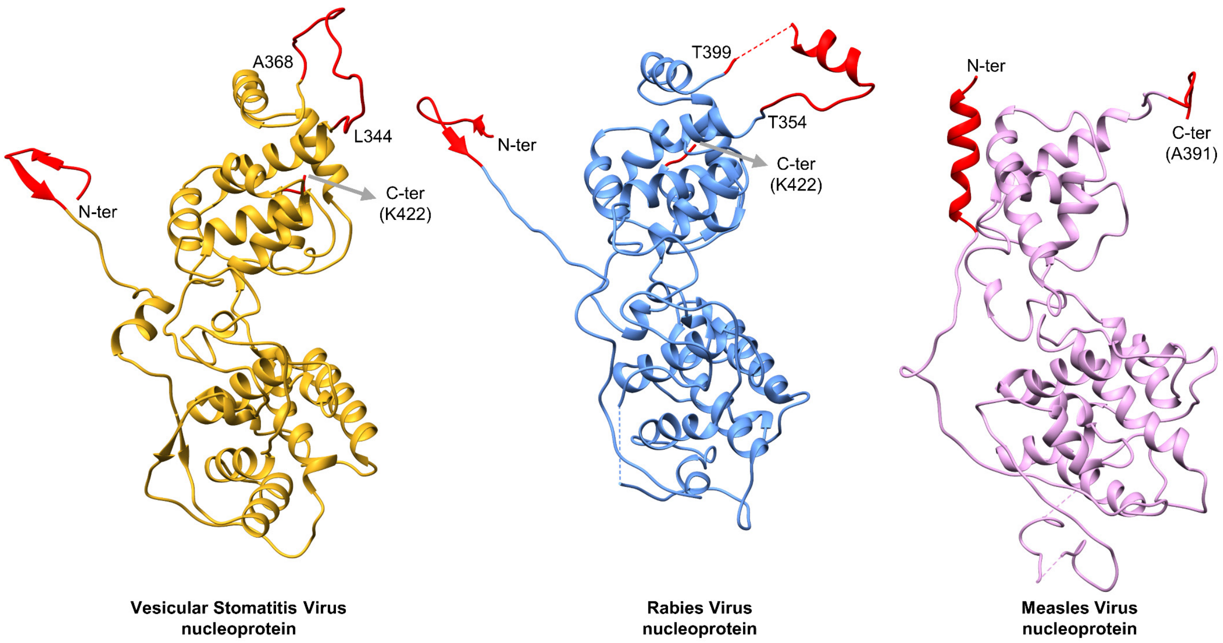

Supplementary Fig. 4 | Structure comparison of nucleoproteins (N) from VSV, rabies virus and measles virus. The N-termini, C-termini and C-loop are highlighted in red.

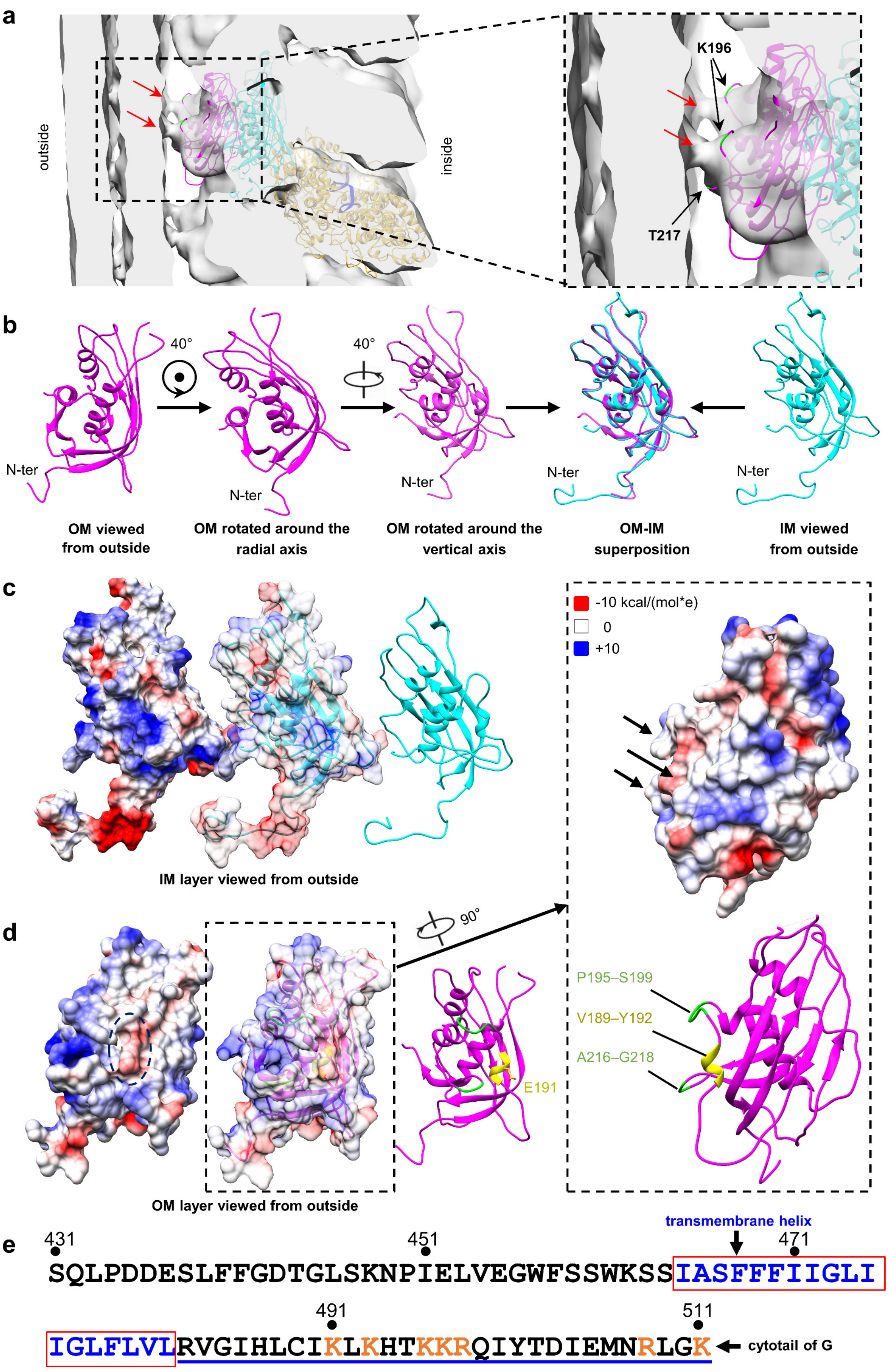
a
outside
inside
K196
T217
b
40°
N-ter
OM viewed from outside
OM rotated around the radial axis
OM rotated around the vertical axis
OM-IM superposition
IM viewed from outside
c
-10 kcal/(mol*e)
0
+10
IM layer viewed from outside
d
90°
E191
P195–S199
V189–Y192
A216–G218
OM layer viewed from outside
e
transmembrane helix
431
451
471
SQLPDDESLFFGDTGLSKNPIELVEGWFSSWKSSIASFFFIIGLI
491
511
IGLFLVLRVGIHLCIKLKHTKKRQIYTDIEMNRLGK
cytotail of G

Supplementary Fig. 5 | Interactions between OM and viral membrane and between OM and G endodomain. a: Subtomogram averaged structure of the nucleocapsid fitted with the atomic models solved above, showing the thin densities linking the OM and the inner leaflet of the viral membrane. Red arrows indicate the linking densities. **b**: Step-by-step superposition between OM and IM subunits. **c**: Outside surface potential of IM. **d**: Outside surface potential of OM. The negatively charged region is marked by dashed lines; a small helix corresponding to this region is colored in yellow. Two loops located close to the membrane are colored in green. **e**: The amino acid sequences of 80 C-terminal residues of G.

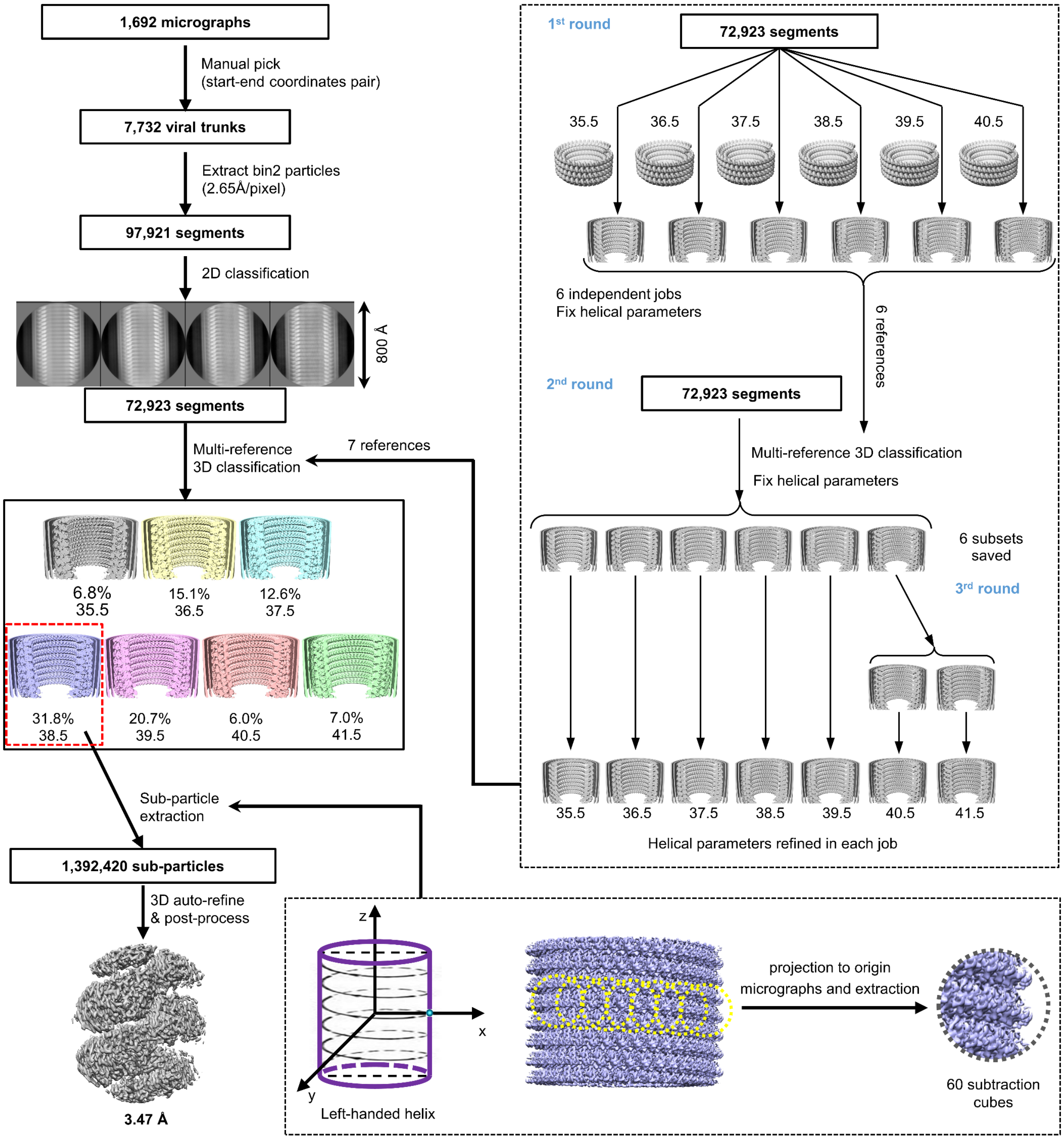

Supplementary Fig. 6 | Data processing workflow for helical sub-particle reconstruction.

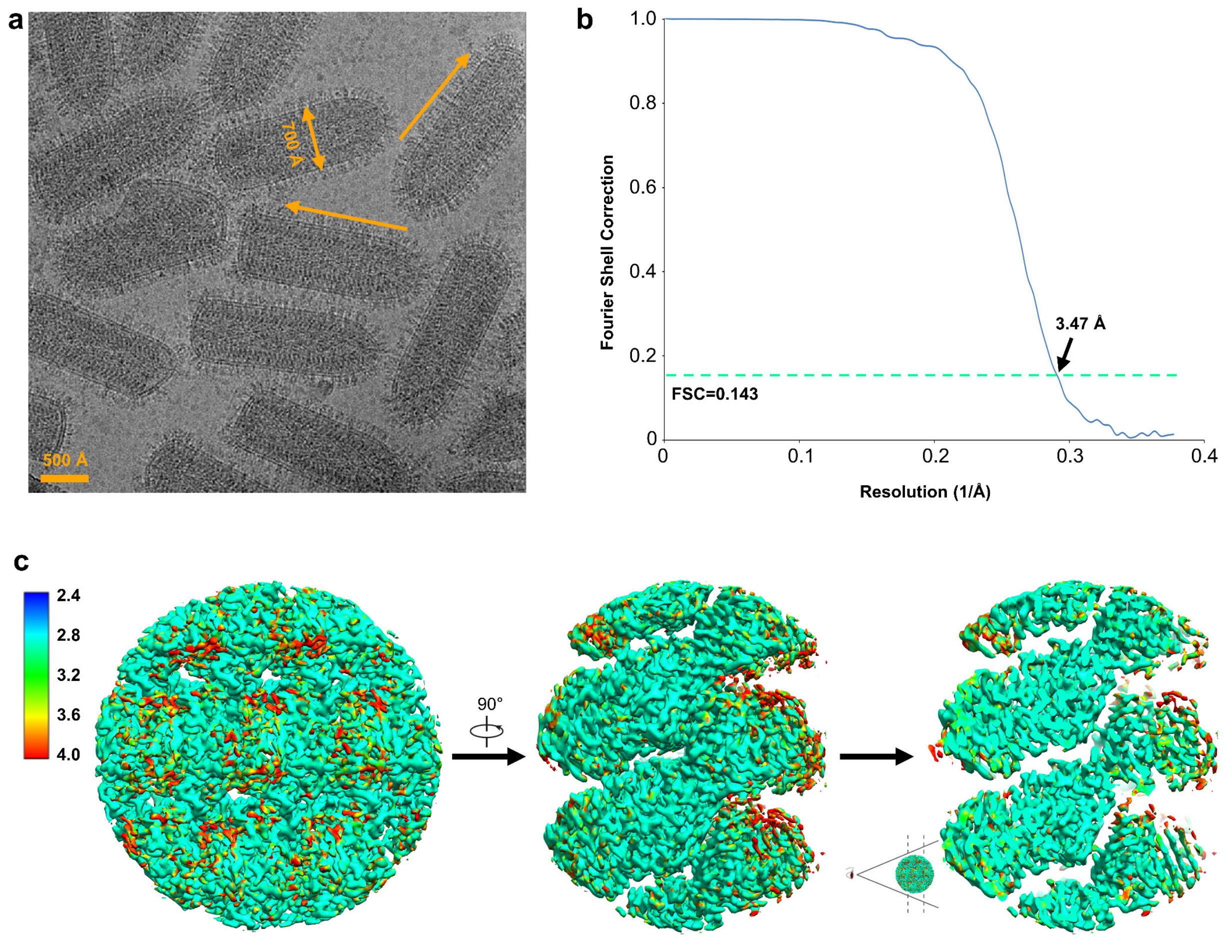

Supplementary Fig. 7 | Near atomic resolution cryoEM analysis for the VSV trunk by helical sub-particle reconstruction. a: A representative cryoEM micrograph of VSV virions from 1692 good micrographs. **b:** Global resolution evaluation based on "gold-standard" Fourier shell correction (FSC) coefficient as a function of spatial frequency generated by RELION, showing a resolution of 3.47 Å based on the 0.143 cut-off of FRC coefficient. **c:** Local resolution evaluation of the reconstructed sub-particle map.

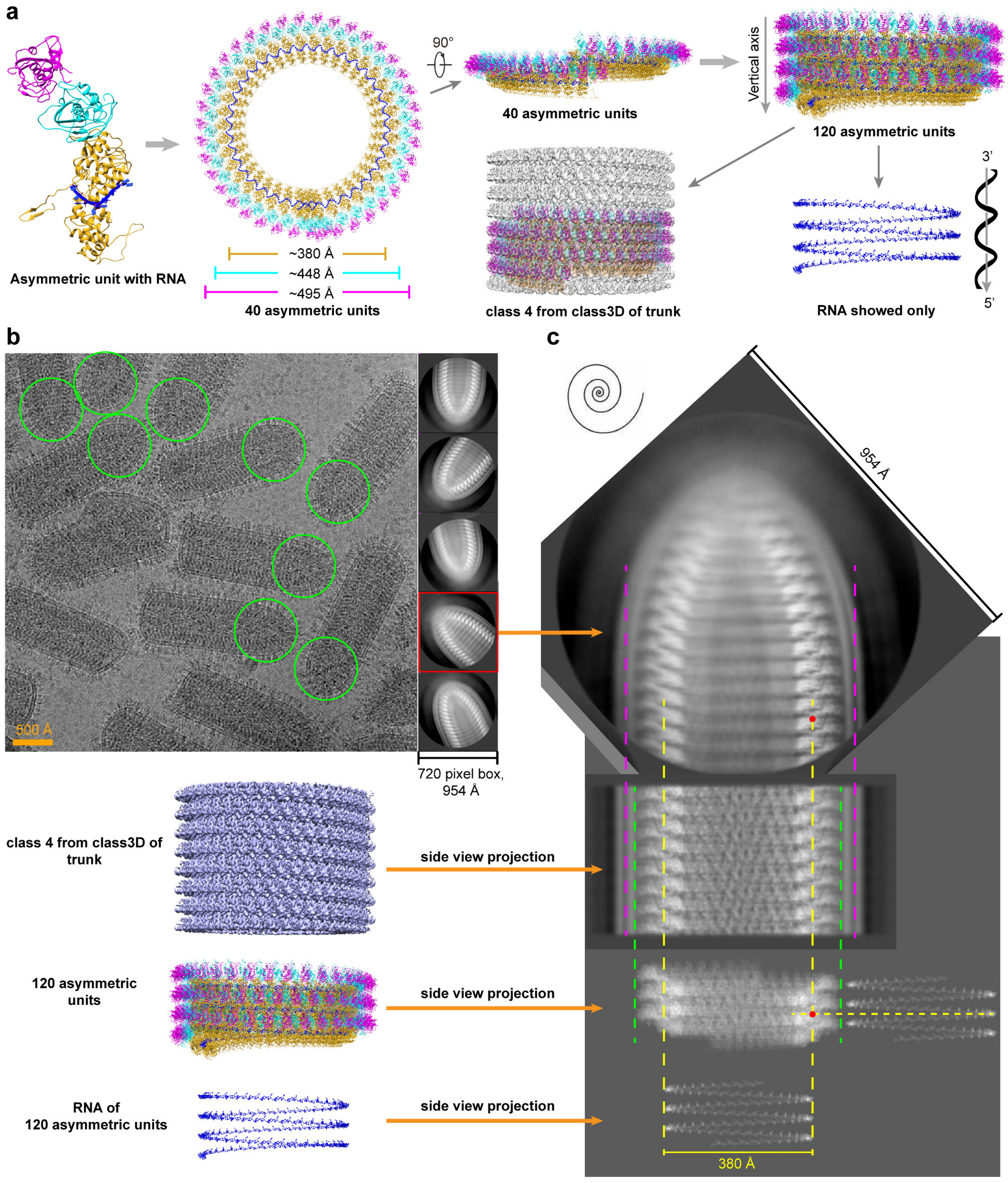

Supplementary Fig. 8 | Preparation of the tip region for capsid reconstruction. a: Helical turns obtained by applying helical symmetry (helical twist: -9.35°, helical rise: 1.3 Å) to one asymmetric unit. The model of 120 asymmetric units fit perfectly in 3D class 4 from helical sub-

particle reconstruction. **b:** A representative cryoEM micrograph of VSV virions from 1692 micrographs and 2D class averages of tips. The tips of virions are indicated by green circles. **c:** Alignment between the tip region and the trunk region. From top to bottom: 2D class of tips, projection of 3D class 4 from helical sub-particle reconstruction of trunk, projection of the 120 asymmetric unit model, and projection of RNA alone. The yellow dashed lines and red dots indicate the location of RNA inside 2D class of tips.

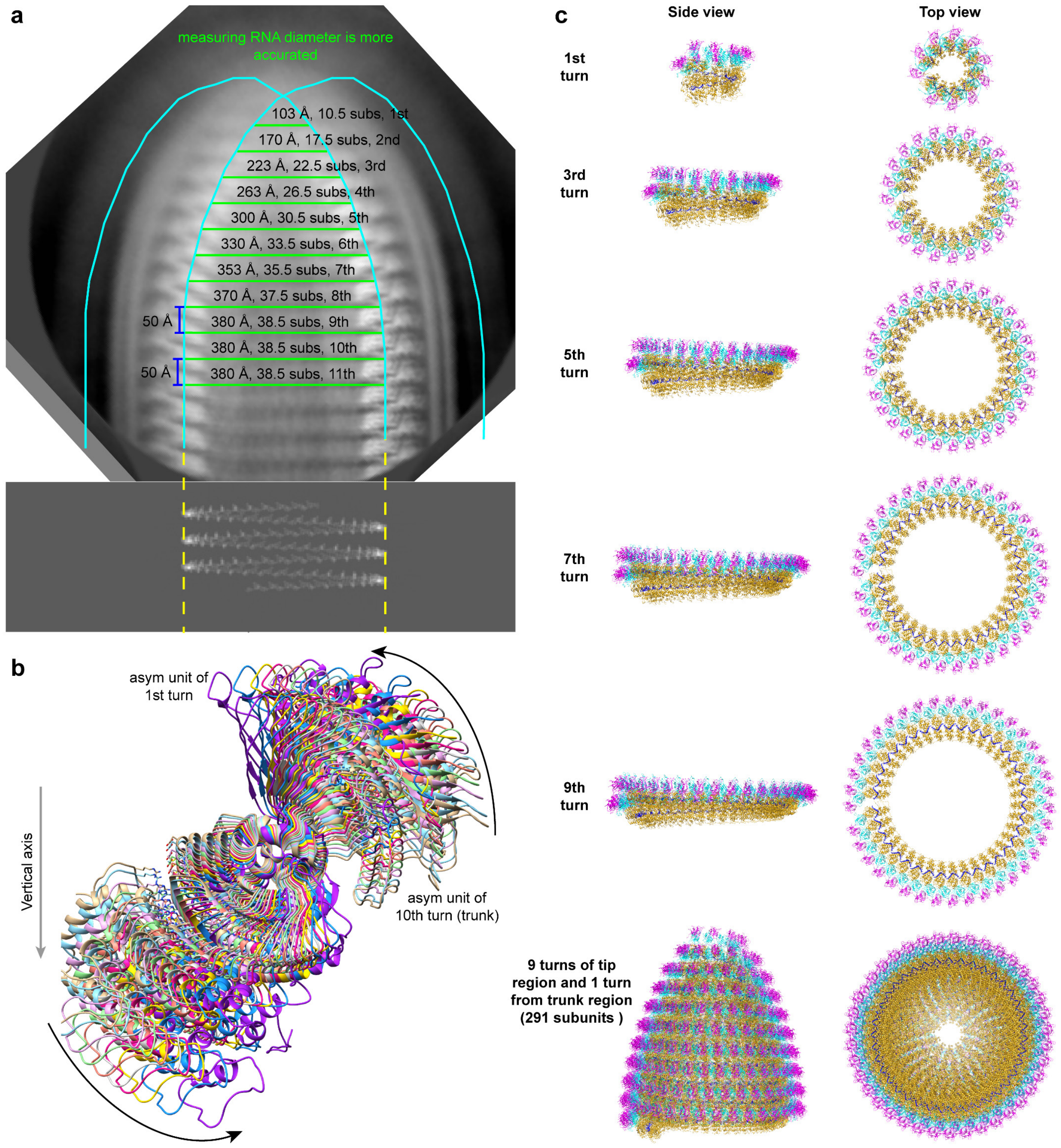

Supplementary Fig. 9 | Tip region reconstruction. a: Definition of the RNA strand edge in the tip region; the diameter of each RNA turn and their corresponding asymmetric unit (sub) numbers. **b:** The asymmetric unit model rotation of each turn in the tip region. **c:** Reconstructed model of turns and the composed model of the tip region.

Supplementary Tables

**Subunit 1**	**Subunit 2**	**Interface area (Å^2)**	**Δ^{i}G (kcal/mol)**
N_U	N_{U+1}	2551.6	-20.8
N_{U-1}	N_{U+1}	321.4	-5.4
N_U	N_L	29.5	0.1
N_U	N_{L+1}	180.8	0.5
IM_L	N_U	451.8	0
**IM_U**	**N_U**	398.9	-4.4
IM_U	N_{U+1}	229.1	-2.3
IM_U	IM_L	413.2	-4.3
**IM_U**	**OM_U**	884.2	-8.5
IM_U	OM_{U-1}	689.5	-2.9

Supplementary Table 1 | Interface area and Δ^{i}G between subunits analyzed by PISA software.

Class number	Helical twist (°)	Helical rise (Å)	Subunits number per turn	Rise per turn (Å)
1	-10.14	1.42	35.50	50.41
2	-9.86	1.38	36.51	50.38
3	-9.60	1.34	37.50	50.25
4	-9.35	1.30	38.50	50.05
5	-9.12	1.27	39.47	50.13
6	-8.89	1.24	40.49	50.21
7	-8.68	1.21	41.47	50.18

Supplementary Table 2 | Helical parameters of 7 classes from 3D classification.

	VSV virion trunk (EMD-26841, PDB 7UWS)
**Data collection and processing**	
Magnification	×105,000
Voltage (kV)	300
Electron exposure (e–/Å^2)	50
Defocus range (μm)	1.5 – 3.0
Pixel size (Å)	1.325
**Helical reconstruction**	
Symmetry imposed	C_1
Initial particle images (no.)	97,921
Final particle images (no.)	23,207
Helical twist (°)	-9.35
Helical rise (Å)	1.30
**Sub-particle reconstruction**	
Symmetry imposed	C_1
Particle images (no.)	1,392,420
Map resolution (Å)	3.47
FSC threshold	0.143
**Refinement**	
Initial model used (PDB code)	2GIC, 2W2R
Model resolution (Å)	3.47
FSC threshold	0.143
Map sharpening B factor (Å^2)	-170
Map CC (mask)	0.78
Model composition	
Non-hydrogen atoms	39,416
Protein residues	4,766
RNA nucleotides	68
B factors (Å^2)	
Protein	74.16
RNA	64.46
R.m.s. deviations	
Bond lengths (Å)	0.003
Bond angles (°)	0.584
Validation	
MolProbity score	1.96
Clashscore	10.97
Poor rotamers (%)	0.07
Ramachandran plot	
Favored (%)	94.01
Allowed (%)	5.99
Disallowed (%)	0

Supplementary Table 3 | CryoEM data collection, refinement and validation statistics.

Turn	RNA cycle diameter (Å)	Subunit/turn	Helical twist (°)	Helical rise (Å)	Subunit rotation angle
1st	103	10.5	-34.29	4.76	--- (40 given)
2nd	170	17.5	-20.57	2.86	31
3rd	223	22.5	-16	2.22	25
4th	263	26.5	-13.58	1.89	21.25
5th	300	30.5	-11.8	1.64	18.75
6th	330	33.5	-10.75	1.49	15
7th	353	35.5	-10.14	1.41	11.25
8th	370	37.5	-9.6	1.33	7.5
9th	380	38.5	-9.35	1.30	2.75
10th (first turn of trunk)	380	38.5	-9.35	1.30	0

Supplementary Table 4 | Calculated parameters of each turn in tip region.
