# Supplementary material for: Atomic model of vesicular stomatitis virus and mechanism of assembly
Source: Nat Commun. 2022 Oct 10;13:5980. doi: 10.1038/s41467-022-33664-4 (PMC9549855; doi:10.1038/s41467-022-33664-4)
Supplement: Supplementary file 3 — Description to Additional Supplementary Information [file 41467_2022_33664_MOESM3_ESM.pdf]

Description of Additional Supplementary Files

Supplementary Movie 1 | Surface view of the sub-particle reconstruction of the VSV trunk. The full density map is displayed as gray, followed by colored segmentations for intact OM (magenta), IM (cyan) and N (orange) with associated RNA (blue). Related to Figure 1.

Supplementary Movie 2 | Ribbon diagram of the atomic model built into the sub-particle reconstruction of the VSV trunk. The model contains 5 OM (magenta) subunits, 7 IM (cyan) subunits and 7 N (orange) subunits and two RNA (blue) fragments. Related to Figure 2.

Supplementary Movie 3 | Slicing through representative tomograms reconstructed by SIRT showing virions with prefusion G trimers and postfusion G trimers.

Supplementary Movie 4 | The reconstructed entire VSV virion. 360° panorama of entire VSV virion. N, IM, OM, RNA, L, P and G are colored in goldenrod, cyan, magenta,blue, red, black and green, respectively. Membrane is colored in gray.

Supplementary Movie 5 | Ribbon diagram of the VSV capsid. Full capsid, partial layers hidden capsid and RNA alone were displayed.
