# Supplementary material for: Atomic model of vesicular stomatitis virus and mechanism of assembly
Source: Nat Commun. 2022 Oct 10;13:5980. doi: 10.1038/s41467-022-33664-4 (PMC9549855; doi:10.1038/s41467-022-33664-4)
Supplement: Supplementary file 9 — Reporting Summary [file 41467_2022_33664_MOESM9_ESM.pdf]

nature research

Corresponding author(s): Z. Hong Zhou

Last updated by author(s): Sep 16, 2022

Reporting Summary

Nature Research wishes to improve the reproducibility of the work that we publish. This form provides structure for consistency and transparency in reporting. For further information on Nature Research policies, see our Editorial Policies and the Editorial Policy Checklist.

Statistics

For all statistical analyses, confirm that the following items are present in the figure legend, table legend, main text, or Methods section.

n/a	Confirmed	
☐	☒	The exact sample size (n) for each experimental group/condition, given as a discrete number and unit of measurement
☐	☒	A statement on whether measurements were taken from distinct samples or whether the same sample was measured repeatedly
☒	☐	The statistical test(s) used AND whether they are one- or two-sided<br>*Only common tests should be described solely by name; describe more complex techniques in the Methods section.*
☒	☐	A description of all covariates tested
☒	☐	A description of any assumptions or corrections, such as tests of normality and adjustment for multiple comparisons
☐	☒	A full description of the statistical parameters including central tendency (e.g. means) or other basic estimates (e.g. regression coefficient) AND variation (e.g. standard deviation) or associated estimates of uncertainty (e.g. confidence intervals)
☒	☐	For null hypothesis testing, the test statistic (e.g. F, t, r) with confidence intervals, effect sizes, degrees of freedom and P value noted<br>*Give P values as exact values whenever suitable.*
☒	☐	For Bayesian analysis, information on the choice of priors and Markov chain Monte Carlo settings
☒	☐	For hierarchical and complex designs, identification of the appropriate level for tests and full reporting of outcomes
☒	☐	Estimates of effect sizes (e.g. Cohen's d, Pearson's r), indicating how they were calculated

Our web collection on statistics for biologists contains articles on many of the points above.

Software and code

Policy information about availability of computer code

Data collection	Leginon 3.1, SerialEM 3.8
Data analysis	Softwares used in data process and analysis: MothinCor 2, Ctffind 4.1.5, Bsoft 1.8.8, emClarity 1.4.3, IMOD 4.9, Resmap 1.1.4, MolProbity v4.2, PISA 2.1, ISONET, Relion 2.0 and 3.0. Atomic models were built and refined using winCOOT 0.8.9 and Phenix 1.18.2. Atomic models and maps were visualized using UCSF Chimera 1.16 and IMOD 4.9.<br>The program helisub.C is provided in Github (https://github.com/EICN-UCLA/helisub).

For manuscripts utilizing custom algorithms or software that are central to the research but not yet described in published literature, software must be made available to editors and reviewers. We strongly encourage code deposition in a community repository (e.g. GitHub). See the Nature Research guidelines for submitting code & software for further information.

Data

Policy information about availability of data

All manuscripts must include a data availability statement. This statement should provide the following information, where applicable:

- Accession codes, unique identifiers, or web links for publicly available datasets
- A list of figures that have associated raw data
- A description of any restrictions on data availability

The sub-particle reconstruction density map generated in this study has been deposited in the Electron Microscopy Data Bank under accession code EMD-26841, the corresponding atomic model is deposited in the Protein Data Bank under the accession code 7UWS (https://doi.org/10.2210/pdb7uws/pdb).The previously published VSV structures used in this study are available in the Protein Data Bank under accession codes 2GIC (http://doi.org/10.2210/pdb2gic/pdb), 2WYY (http://doi.org/10.2210/pdb2wyy/pdb) and 2W2R (http://doi.org/10.2210/pdb2w2r/pdb). The pseudo-atomic model of the entire VSV virion generated in this study is publicly available from the corresponding author.

Field-specific reporting

Please select the one below that is the best fit for your research. If you are not sure, read the appropriate sections before making your selection.

☒ Life sciences ☐ Behavioural & social sciences ☐ Ecological, evolutionary & environmental sciences

For a reference copy of the document with all sections, see nature.com/documents/nr-reporting-summary-flat.pdf

Life sciences study design

All studies must disclose on these points even when the disclosure is negative.

Sample size	For cryoEM analysis, 1,692 images (72,923 segments) were used for 3D classification of VSV trunk. For the subset having 38.5 asymmetric units per turn, 1,392,420 sub-particles obtained from 23,207 segments were used for sub-particle reconstruction. For cryoET analysis, 30 tilt series and 26 tilt series were acquired to get G in postfusion and prefusion conformations, respectively. After the virions classification, 6030 postfusion G trimers from 57 virions having 38.5 asymmetric units per turn and 4452 prefusion G trimers from 39 virions having 38.5 asymmetric units per turn were contributed to the averaged density map.
Data exclusions	For cryoEM analysis, 316 micrographs were excluded owing to the bad qualities. During 2D classification, particles that do not belong to the class of interest or have poor qualities were excluded. For CryoET analysis, 20 tilt series and 11 tilt series were excluded from the two batches, respectively, due to bad quality or the absence of virions with 38.5 asymmetric units per turn.
Replication	For cryoEM dataset, all data in this work were collected from one cryo grid. For cryoET data collection, the cryo grids were prepared with three independently prepared batches of samples, and the results keep consistent. The results presented in this paper are from one batch of sample.
Randomization	During the 3D auto-refinements for both cryoEM and cryoET analysis, each dataset was randomly split to two half for calculating the Fourier Shell Correlation (FSC).
Blinding	Blinding was not feasible to this study. Since we are investigating a specific virus, there was no blinding in data collection and structural analysis.

Reporting for specific materials, systems and methods

We require information from authors about some types of materials, experimental systems and methods used in many studies. Here, indicate whether each material, system or method listed is relevant to your study. If you are not sure if a list item applies to your research, read the appropriate section before selecting a response.

Materials & experimental systems

n/a	Involved in the study	
☒	☐	Antibodies
☐	☒	Eukaryotic cell lines
☒	☐	Palaeontology and archaeology
☒	☐	Animals and other organisms
☒	☐	Human research participants
☒	☐	Clinical data
☒	☐	Dual use research of concern

Methods

n/a	Involved in the study	
☒	☐	ChIP-seq
☒	☐	Flow cytometry
☒	☐	MRI-based neuroimaging

Eukaryotic cell lines

Policy information about cell lines

Cell line source(s)	Hela cells
Authentication	Not authenticated.
Mycoplasma contamination	Not tested.
Commonly misidentified lines (See ICLAC register)	Not listed in the commonly misidentified lines.
